# Unexpected Effects of Local Management and Landscape Composition on Predatory Mites and Their Food Resources in Vineyards

**DOI:** 10.3390/insects12020180

**Published:** 2021-02-19

**Authors:** Stefan Möth, Andreas Walzer, Markus Redl, Božana Petrović, Christoph Hoffmann, Silvia Winter

**Affiliations:** 1 Institute of Plant Protection, University of Natural Resources and Life Sciences Vienna (BOKU), Gregor-Mendel-Straße 33, 1180 Vienna, Austria; andreas.walzer@boku.ac.at (A.W.); markus.redl@boku.ac.at (M.R.); bozana.petrovic@boku.ac.at (B.P.); silvia.winter@boku.ac.at (S.W.); 2 Julius Kühn-Institute (JKI), Institute for Plant Protection in Fruit Crops and Viticulture, Geilweilerhof, 76833 Siebeldingen, Germany; christoph.hoffmann@julius-kuehn.de

**Keywords:** Phytoseiidae, Tydeidae, Eriophyidae, biological pest control, viticulture, cover crops, pollen, pesticide toxicity index

## Abstract

**Simple Summary:**

Sustainable agriculture becomes more important for biodiversity conservation and environmental protection. Viticulture is characterized by relatively high pesticide inputs, which could decrease arthropod populations and biological pest control in vineyards. This problem could be counteracted with management practices such as the implementation of diverse vegetation cover in the vineyard inter-rows, reduced pesticide input in integrated or organic vineyards, and a diverse landscape with trees and hedges. We examined the influence of these factors on predatory mites, which play a crucial role as natural enemies for pest mites on vines, and pollen as important alternative food source for predatory mites in 32 organic and integrated Austrian vineyards. Predatory mites benefited from integrated pesticide management and spontaneous vegetation cover in vineyard inter-rows. Pest mite populations were very low and sometimes completely absent on vines. This showed that agri-environmental schemes should consider less intensive pesticide use and spontaneous vegetation cover in the vineyard inter-row due to the beneficial effect on predatory mite populations and their related biological control potential in vineyards.

**Abstract:**

Viticultural practices and landscape composition are the main drivers influencing biological pest control in vineyards. Predatory mites, mainly phytoseiid (Phytoseiidae) and tydeoid mites (Tydeidae), are important to control phytophagous mites (Tetranychidae and Eriophyidae) on vines. In the absence of arthropod prey, pollen is an important food source for predatory mites. In 32 paired vineyards located in Burgenland/Austria, we examined the effect of landscape composition, management type (organic/integrated), pesticide use, and cover crop diversity of the inter-row on the densities of phytoseiid, tydeoid, and phytophagous mites. In addition, we sampled pollen on vine leaves. *Typhlodromus pyri* Scheuten was the main phytoseiid mite species and *Tydeus goetzi* Schruft the main tydeoid species. Interestingly, the area-related acute pesticide toxicity loading was higher in organic than in integrated vineyards. The densities of phytoseiid and tydeoid mites was higher in integrated vineyards and in vineyards with spontaneous vegetation. Their population also profited from an increased viticultural area at the landscape scale. Eriophyoid mite densities were extremely low across all vineyards and spider mites were absent. Biological pest control of phytophagous mites benefits from less intensive pesticide use and spontaneous vegetation cover in vineyard inter-rows, which should be considered in agri-environmental schemes.

## 1. Introduction

Plant diversity at the local and landscape scale plays a crucial role for herbivorous pest species, natural enemies, and their interactions in agroecosystems [1,2,3]. Large crop monocultures in simple landscapes could promote pest outbreaks because of two main reasons: (1) food is not a limiting resource for herbivores, specialized to feed on the crop species in monocultures (i.e., resource concentration hypothesis), and (2) the lack of alternative food resources, oviposition sites, overwintering locations, and shelter for natural enemies hinder their permanent establishment in monocultures (i.e., natural enemy hypothesis) [2,4,5,6]. Thus, the nearly unlimited food resources and the lack of natural enemies constitute ideal conditions for specialized herbivores in such agroecosystems. In contrast, pest abundance might also be dependent on non-crop habitats as alternative food resources or for overwintering [7], which could limit the positive feedback of simple landscapes for pest outbreaks.

However, studies evaluating the natural enemy hypothesis [4,5] have provided divergent results—high plant diversity increased the abundance of natural enemies, resulting in lower pest densities in peach orchards and cacao agroforestry systems [8,9], whereas such positive plant diversity effects were not observed in annual crops such as squash and cabbage [10,11].

Along the same line, the predictions of the resource concentration hypothesis [4,5] were verified for the cereal leaf beetle [12], the turnip root fly [11], and the potato leafhopper [13], but not for the squash bug [10] or the Andean potato weevil [14]. The effects of large monocultures and alternative habitats at landscape scale on pests and their natural enemies seem to differ with regard to the respective crop system and the pest species [15]. One reason for the diverging results could be related to pest stochasticity, which could mask the positive effects of landscape complexity on pest outbreaks [16].

Viticulture is a perennial, monoculture crop system distributed on all continents but Antarctica [17], and is found in Mediterranean, oceanic, continental, and steppe climates [18]. In contrast to arable crops, vineyards are characterized by relatively large non-productive inter-rows whose vegetation may constitute diverse ecosystems [19,20]. The vegetation composition can vary greatly depending on vineyard management (differing in tillage intensity, herbicide use, mowing frequency, different cover crop mixtures, or spontaneous vegetation), soil type, and climatic conditions [20,21]. Vegetation management of vineyard inter-rows clearly influence biodiversity and ecosystem service provision in vineyards [22,23,24]. Decreasing management intensity with temporary or permanent vegetation cover in the inter-rows increases plant species richness [19,20], which is beneficial for insect–flower interactions [25] and results in diverse arthropod populations [26,27,28,29]. Native cover crops in particular enhance plant-dwelling arthropod diversity in vineyards compared to exotic cover crops [30].

Different pest species may significantly reduce grape quality and quantity beyond economic thresholds [31]. Therefore, biological control is an important factor to promote sustainable viticulture [17] and decrease the use of insecticides and acaricides in order to achieve the EU policy goal of halving pesticide use by 2030 [32]. It has been shown that landscape composition influences the effectiveness of natural enemies of some pest species. Diverse landscapes have reduced infestation of grape berry moth and insecticide applications in Spanish vineyards [16]. Similarly, mealybug and mite infestation decreased when the proportion of semi-natural habitats (SNHs) increased in the landscape [33]. Furthermore, surrounding SNHs harbor phytoseiid mites [34], which could disperse into nearby vineyards [35].

Spider mites (Acari: Tetranychidae) and eriophyoid mites (Acari: Eriophyidae) are important phytophagous mites in viticulture that can create problems in vineyards due to the loss of grape yield and quality [36]. The damage caused by spider mites are, e.g., leaf discoloration, loss of chlorophyll, and leaf drop, whereas eriophyoid mites cause, e.g., death of overwintering buds, blisters on vine leaves, and the development of lateral shoots [36]. Currently, there are no severe problems regarding phytophagous mites in Austrian viticulture. Compared to spider mite outbreaks in the second half of the 19th and 20th centuries, pest mite populations are nowadays often low in European vineyards, due to the biological control by predators such as phytoseiid mites (Acari: Phytoseiidae) [36]. Furthermore, some tydeoid mite species (Acari: Tydeidae) also prey on eriophyoid mites [37,38]. The successful establishment of stable generalist predatory mite populations on vines is important to maintain effective biological pest control, which is also dependent on alternative food resources such as other mite species [39] or pollen and fungi [39,40,41]. The nutritive species-specific value of pollen differs in relation to development and reproduction of phytoseiid mite species [42,43]. Pollen is also an important food source for tydeoid mites [44,45]. Inter-row vegetation [23] at the local scale and SNHs at the landscape scale provide these pollen resources during the vegetation period [46,47].

Natural enemy populations are strongly affected by pesticide use. Organic vineyards were found to exhibit higher population densities and species richness of phytoseiid mites compared with conventional vineyards in Italy [48] and Portugal [49]. Along the same line, organic vineyards had a higher abundance of arthropod predators compared to conventional vineyards in Italy [50] and also showed a higher and stable predation rate of the grape berry moth in France [51]. In organic viticulture, only inorganic and bio-pesticides are permitted (but no herbicides), whereas in conventional viticulture, a broad range of also synthetic pesticides can be applied [50]. It is considered that the choice of pesticides, their calculated toxicity loads [52], and the treatment frequency are major factors for natural enemy populations, including phytoseiid mites [53,54]. The treatment frequency index (TFI) does not take the toxicity of the sprayed products towards non-target organisms into account [33,55,56,57] and is related to the recommended product-specific dose, which could differ between European countries due to their division into climatic zones during the registration process of plant protection products [58]. The acute insecticide toxicity loading (AITL) tries to reflect toxicity loads on the basis of LD_50_ values for honeybees and environmental persistence of the active ingredients [59]. As this index is not related to the sprayed area, an area-related index needs to be calculated (for details, see Section 2.6. in the Material and Methods).

To the best of our knowledge, this is the first scientific article investigating the effects of two local management practices (pesticide and ground cover management) and landscape composition on predatory mites and their food sources (phytophagous mites and pollen) in vineyards. Furthermore, we calculated a novel area-related toxicity index for organic and integrated vineyards and compared it with another pesticide intensity index. Three main questions guided this article: (i) Which field or landscape scale parameters show the strongest effect on the densities of phytoseiid, tydeoid, and phytophagous mites? (ii) Does organic management, reduced pesticide input, or the use of species-rich cover crops increase predatory mite density and diversity? (iii) Does landscape diversity and a higher proportion of SNHs increase pollen availability, diversity, and consequently also phytoseiid mite densities in vineyards?

## 2. Materials and Methods

### 2.1. Study Sites

**Figure 1:**
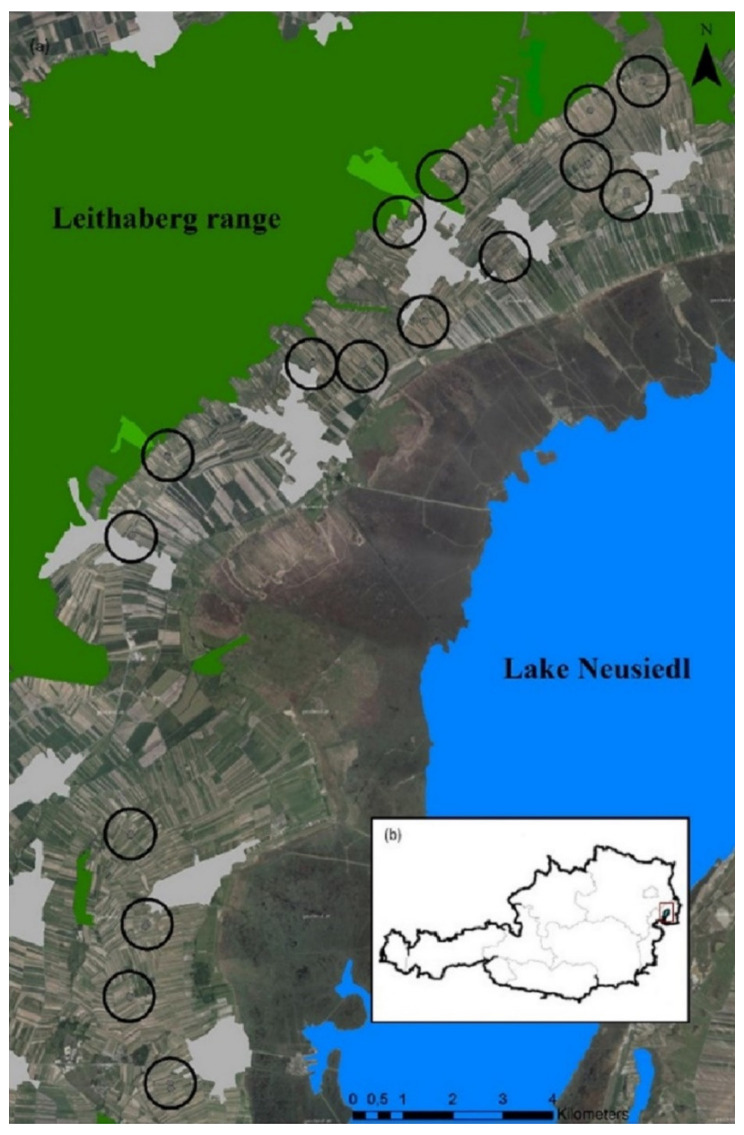
Image of the study area: (**b**) study area located in eastern Austria at the Neusiedlersee-Hügelland (Leithaberg); (**a**) aerial image [61] with the location of the vineyards including woods (green) and the lake Neusiedl (blue). Each circle (diameter of 500 m) includes two paired vineyards differing in management type (integrated versus organic).

Overall, we selected 32 commercial vineyards, located in Burgenland (Austria) in the wine-growing region Leithaberg (47°54′55.2754″ N; 16°41′40.4453″ E) (Figure 1). The climate is warm temperate [18], the 10-year (2010–2019) average temperature was 11.6 °C, and total annual precipitation was 574 mm [60]. This region is characterized by a small-scaled mosaic of SNHs, vineyards, and arable fields. SNHs include meadow orchards, dry grasslands, fallows, woods, single trees, and hedges. Vineyards are situated on plain or hilly terrain and their scale is typically small (0.25–1.5 ha), long, and narrow, consisting only out of a few rows managed with the trellis system (2.25 × 0.9 m) with a trunk height of about 0.9 m. Vineyards are planted with a wide range of different white and red grapevine varieties.

Vineyards were selected according to three different criteria: (i) pairs of vineyards (maximum distance of 200 m) differing according to the **farming system**: organic and integrated vineyards (integrated vineyards are characterized by the implementation of integrated pest management practices with a focus on preventive measures to reduce the use of pesticides by considering the threshold of damage principle and by supporting natural enemies [17,36]) (see Table S1 for further information on applied active ingredients of organic and integrated vineyards); (ii) **inter-row cover crop type**: high diversity (20–34 species seeded), low diversity (4–9 species) cover crop mixtures, and spontaneous vegetation cover (no seeding of cover crops for at least 5 years); and (iii) the **surrounding landscape composition** differing in the proportion of SNHs, vineyards, and total agricultural area within a 500 m radius around each studied vineyard. Details on individual vineyard management practices (e.g., frequency and timing of tillage operations or pesticide applications, age of the vineyards) were gathered by personal interviews with all winegrowers on the basis of a structured questionnaire in 2019. We noted that during these interviews the winegrowers mentioned that *Typhlodromus pyri* Scheuten was released at a large scale at the Leithaberg vineyards around the 1990s and 2000s due to problems with spider mites. These applications were performed through an inoculation of the vineyards with textile strips or cut vine twigs from a donor vineyard, which already harbored *T. pyri*.

### 2.2. Mite Sampling

Mite sampling was conducted 5 times between May and August 2019 (7 May, 3 June, 1 July, 29 July, and 26 August) along a 30 m transect in each vineyard. The mites were sampled on vine leaves according to the protocol from Boller [62] and Hill and Schlamp [63] by randomly collecting 25 vine leaves from the whole canopy along the transect. The leaves were stored overnight in a refrigerator at +4 °C in a 2 L container that was filled with water and 1 mL of detergent. The leaves were then washed through a sieve tower with a 630 µm mesh sieve for the rough parts; 63 µm for predator, spider, and tydeoid mites; and 32 µm for the eriophyoid mites. The content from the second and third sieve were each washed onto filter paper with a Büchner funnel and dyed with methylene blue (Methylene blue, Merk, Darmstadt, Germany) and diluted 1:3 with tap water. The abundance was counted with a stereomicroscope (Leica M80, Wetzlar, Germany) at 25× magnification for phytoseiid, tydeoid, and spider mites at the family level [64,65]. Eriophyoid mites were counted at species level with 50× magnification [64,66]. Furthermore, phytoseiid and tydeoid mites were selected from the filter paper and mounted in Hoyer’s medium [67], and then species identification was carried out with a light microscope (Zeiss Axioplan 2 imaging, Oberkochen, Germany) at 200× and 400× magnification and phase contrast. Phytoseiid mites were identified after the identification key for Phytoseiidae from Tixier et al. [68] and tydeoid mites were identified with the key for Tydeidae from Da Silva et al. [37]. The leaf area of the vine leaves was measured after the extraction of the mites with a Li-COR Modell 3100 area meter (Lincoln, NE, USA) to calculate mite density per 100 cm^2^.

### 2.3. Pollen Sampling

Pollen samples were collected in parallel to the mite samples 5 times along the same 30 m transect. Per vineyard, 15 randomly chosen samples were collected according to the method from Addison et al. [69]. A 5 cm long stripe of invisible sticky tape was adhered onto the upper surface of grapevine leaves at the beginning of the petiole and centered on the mid-rib. A 19 × 19 mm square was cut out from each stripe and mounted with a glycerine (Glycerin Rotipuran, Roth, Karlsruhe, Germany) and fuchsin solution (Fuchsine basic (C.I. 42510) Roth, Karlsruhe, Germany) on slides to increase the contrast of the pollen grains [70]. The pollen grains were identified at family level with a key from Beug [71] and a light microscope (Nikon Optiphot 2, Tokyo, Japan) at 400× magnification. Due to similar morphological characteristics of the pollen grains, we merged the pollen types Amaranthaceae with Caryophyllaceae, and Moraceae were merged with Urticaceae. When it was not possible to identify the pollen grains at family level, we classified them as arboreal pollen (AP) or non-arboreal pollen (NAP). Non-identifiable pollen was categorized as NA (not able to identify). To reach a representative subsample, we used the method from Rossi et al. [72], counting the pollen grains with 200× magnification (Nikon Optiphot 2, Tokyo, Japan) on 4 equidistant transects [72], which were then extrapolated to pollen grain numbers per square centimeter.

### 2.4. Vegetation Survey

Vegetation surveys in the inter-rows of the vineyard transects were performed in spring (April) and summer (June–July) in four 1 m^2^ plots with approximately 6 m spacing between them. In each plot, the vegetation cover was recorded as a percentage according to the scale of Londo [73] (total vegetation cover). Vegetation cover data in spring and summer were then used for further analysis.

### 2.5. Landscape Survey

Landscape mapping was based on the EUNIS habitat type classification scheme [74] within a 500 m radius around each studied vineyard. This scale was selected on the basis of previous natural pest control studies of the project team and other studies (e.g., [75,76]). In addition, this radius also covers the distance for the aerial dispersal of different phytoseiid mite species ranging between 10 and 200 m [77,78]. Landscape configuration was mapped in the field on the basis of the existing information of the land utilization mapping from Burgenland, Austria [61], and was later digitized and analyzed with ArcGIS [79] and RStudio [80] with the R package “landscapemetrics” [81]. The landscape was aggregated into 14 habitat classes and summarized into 4 landscape parameters as percentages: proportion of woody SNHs (hedgerow, solitary trees, tree rows, and woodland), total SNHs (fallow, grassland, grass strip from field margins, and woody SNHs), vineyards, and total agriculture (vineyards and arable crops). Four additional habitat classes (artificial and constructed entities, ponds and rivers, roads, and yards) were not included in the statistical analysis. Additionally, we computed the minimum distance to the next woody SNHs (in m) and the Shannon’s landscape diversity index (SHDI) as explanatory variables for statistical modelling (Table S2). The parameter woody SNHs and distance to the next woody SNHs were calculated because phytoseiid mites could disperse from woody margins into vineyards [35].

### 2.6. Area-Related Acute Pesticide Contact Toxicity Loading (aAPTLc)

The spraying regimes of each vineyard built the basis for the calculation of our area-related acute pesticide contact toxicity loading (aAPTLc) for each vineyard, modified after DiBartolomeis et al. [59] with a difference in that we used a hazard quotient (HQ) for honeybees [82] for all used pesticides in order to refer to the field area (ha) and a different scaling factor, with the formula

$$aAPTLc=\overset{n}{\underset{i=1}{\sum }}x_{i}\left(\frac{amount\;of\;applied\;active\;ingredient\;\left(g/ha\right)}{honey\;bee\;contact\;LD_{50}\;\left(µg/bee\right)}\right)\times \left(\frac{half-life\;\left(days\right)}{\ln2}\right)\times \;10^{-4}$$

The number of applied active ingredients is indicated with *n*. Honeybee contact LD_50_ and half-life values (soil degradation in days) for each applied active ingredient were obtained through the Pesticide Properties Database (PPDB) [83] and the Bio-Pesticides Database (BPDB) [83]. The amount of the applied active ingredient (g/h) was calculated by multiplying the concentration (%) of the active ingredient for each pesticide product [84] with the applied dose of the product (g) at the field level (ha). A scaling factor of 10^−4^ was chosen for a better visual comparison of the aAPTLc values. Four (out of 38) active ingredients with missing values were excluded from the calculation. As we wanted to include the frequently applied potassium bicarbonate (especially in organic vineyards) for the aAPTLc calculation, despite the missing half-life value, we assumed the half-life of 7 days on the basis of expert knowledge and a registration report draft of a potassium bicarbonate product [85].

We could not calculate an aAPTLc on the basis of predatory mites due to lacking information of LR_50_ values for *T. pyri* for too many applied active ingredients (12 LR_50_ values out of 38 were missing). Therefore, we compared the aAPTLc with a specific categorical toxicity rating for phytoseiid mites. The categorical toxicity rating of the spraying regimes from all vineyards was calculated for the species *T. pyri* after Hassan [86]. For the calculation, we used the maximum harmful effect of the used active ingredients with a minor modification regarding the numerical scaling (scaling: harmless (<40% decrease of beneficial capacity) = 0.4, slightly harmful (40–80% decrease of beneficial capacity) = 0.8, and harmful (>80% decrease of beneficial capacity) = 1) according to the classification of the German plant protection register [87,88]. The categorical toxicity rating for *T. pyri* was then calculated for each vineyard through summing up the numerical scaled values from all applied active ingredients.

### 2.7. Data Analysis

**Figure 2:**
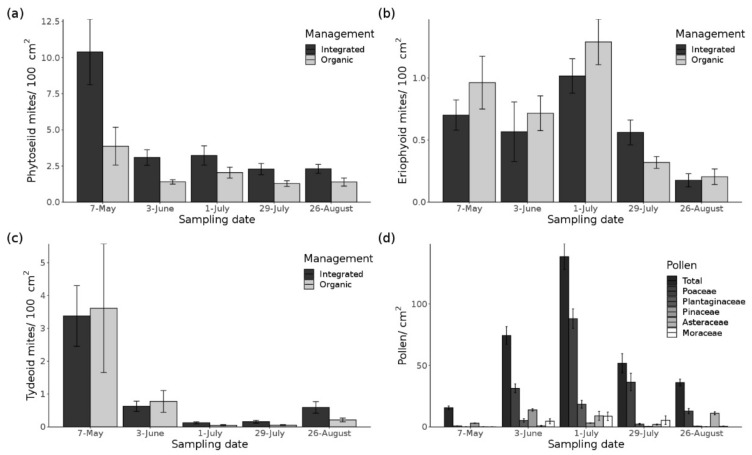
Densities in different scales (mean ± standard deviation (SD)) from 2019 of (**a**) phytoseiid, (**b**) eriophyoid, and (**c**) tydeoid mites per 100 cm^2^ vine leaf area for each sampling date split between integrated and organic vineyards. Densities (mean ± SD) of (**d**) pollen per square centimeter on vine leaves per sampling date with the total pollen and split into five most abundant pollen types. The pollen type Moraceae was merged with Urticaceae due to similar morphological characteristics.

The statistical analysis and visualization was performed with R version 3.6.3 [89] and R Studio [80] including the R package “FD” [90], “lattice” [91], “effects” [92,93], “stats” [89], “corrplot” [94], “lme4” [95], “MuMIn” [96], and “ggplot2” [97]. The densities of eriophyoid mite species were very low, and thus they were merged for each sampling date prior to statistical analysis. Exploration of the data (detection of outliers, homogeneity of the variance, collinearity, and distribution of the response variable) were performed according to Zuur et al. [98]. Due to the data type, detected outliers, and non-normal distribution of the residuals of the response variables, we transformed the data with log_10_ and before transformation, the value 1 was added (log_10_(y + 1)) [99,100,101]. The transformed data were only used for modelling but not for Figure 2. Explanatory variables with a collinearity of cor ≥ ±0.5 were not included in the same model. Generalized linear models (GLM with Gaussian distribution) [89] were chosen instead of the generalized linear mixed models (GLMM) because the inclusion of the random effect in the GLMMs—paired vineyards nested within landscape circles—resulted in very low variances, estimated at zero for the random effect, which leads to possible boundary problems in most models [102]. GLMs were computed to analyze which date, field (vineyard management, cover crops, aAPTLc, total number of pesticide applications (one application can consist of several different pesticides on the same application date), vegetation cover in spring and summer (Table 1)), and/or landscape parameters (proportion of SNHs, proportion of woody SNHs, proportion of vineyards, distance to nearest woody SNHs) affected phytoseiid, tydeoid, and eriophyoid mites and pollen densities. Additionally, for phytoseiid mites, the densities of tydeoid and eriophyoid mites and pollen were also included in the modelling process and for eriophyoid mites, phytoseiid mites were included. Furthermore, to measure the independency of predictors, we calculated the variance inflation factor (VIF) with the package “car” [92]. When VIF > 5, explanatory variables and their interactions were removed from GLMs due to high collinearity between explanatory variables [98,103]. For each variable, a set of 42 models were formulated by combining the non-collinear explanatory variables that were previously mentioned.

The selection of the most parsimonious model was based on the use of the second order Akaike Information Criterion corrected for small sample size (AIC_c_) [104] from the package “AICcmodavg” [105]. We used a minimum difference between the most parsimonious models of Δi of 2 [106] to select the most parsimonious models [107].

Correlations between the aAPTLc and phytoseiid mite densities in relation to the sampling date, categorical toxicity rating for *T. pyri* and the phytoseiid mite densities, the total aAPTLc, and the categorical toxicity rating for *T. pyri* were tested with Spearman’s correlation [108,109] and the R package “psych” [110].

The pollen diversity data were split into 2 datasets corresponding to spring (sampling dates: 7 May and 3 June) and summer (sampling dates: 1 July, 29 July, and 26 August). A non-metric multidimensional scaling (NMDS) with the R package “vegan” [111] was chosen to examine main gradients of variation and show patterns of similarities between pollen communities on grapevine leaves of the investigated vineyards differing in cover crops [112]. The stress value was used to indicate a possible danger of false interpretation of the plots [113]. Field parameters (cover crop type; plant cover in spring and summer; and aggregated cover of Amaranthaceae, Asteraceae, Plantaginaceae, and Poaceae), landscape parameters (SHDI, minimum distance to next woody SNHs, proportion of SNHs, and proportion of total agriculture), and densities of phytoseiid mites were fitted onto the NMDS for the visualization of possible relations of important traits regarding to the community composition [114].

## 3. Results

### 3.1. Phytoseiid Mites

**Figure 3:**
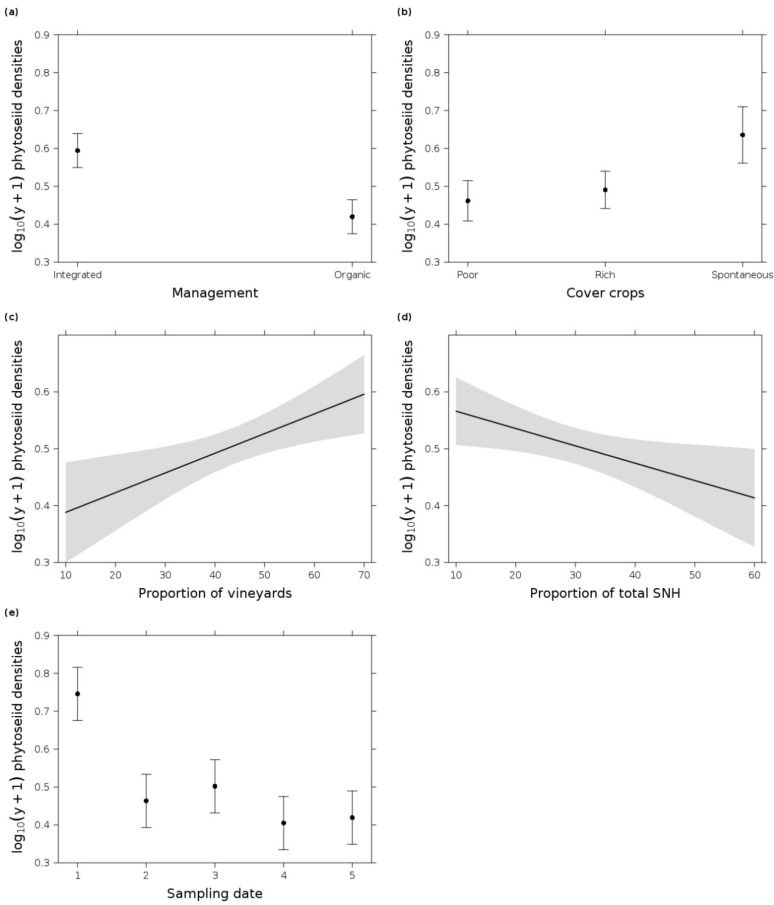
Effect plots of log_10_(y + 1) phytoseiid mite densities per 100 cm^2^ vine leaf area in response to (**a**) management (organic and integrated), (**b**) cover crop type (poor = species-poor, rich = species-rich cover crop mixture, and spontaneous = spontaneous vegetation), (**c**) proportion of vineyards in the landscape, (**d**) proportion of total semi-natural habitats (SNHs) in the landscape, and (**e**) sampling date in 2019 (1 = 7 May, 2 = 3 June, 3 = 1 July, 4 = 29 July, and 5 = 26 August) from the most parsimonious GLM (date + management + cover crop type + proportion of vineyards + proportion of total semi-natural habitats (SNHs)). Error bars/gray shading indicate 0.95 confidence intervals.

The overall mean phytoseiid mite density per 100 cm^2^ vine leaf area was 7.13 ± 7.98 SD (standard deviation) at the beginning of the sampling season, which decreased to 1.85 ± 1.25 at the last sampling date (split into organic and integrated in Figure 2). Out of 6912 sampled phytoseiid mite individuals, we identified three different species with a clear dominance of *T. pyri* (98.73% of all sampled phytoseiid mites), followed by *Euseius finlandicus* (Oudemans) (0.65%) and *Paraseiulus talbii* (Athias-Henriot) (0.62%). Consequently, statistical analysis focused only on the phytoseiid mite densities due to the low overall species richness. Phytoseiid mite populations were present in all vineyards throughout the growing season. The most parsimonious GLM (Table 2) showed that field and landscape parameters affected phytoseiid mite densities over time. The highest phytoseiid mite densities were recorded at the first sampling date with more than twice as many individuals than at other sampling dates (Figure 2). Integrated vineyards had higher phytoseiid mite densities than organic vineyards. Additionally, vineyards with spontaneous vegetation cover exhibited higher phytoseiid mite densities than vineyards with cover crop mixtures. Regarding the landscape parameters, phytoseiid mite densities increased with higher proportion of vineyards at the landscape scale. Vice versa, the increase of SNHs led to a decrease of these mites (Figure 3). Animal food sources (phytophagous and tydeoid mites) did not improve model fit; pollen abundance was only included in the second most parsimonious model (slightly outside the Δi range, Δi = 2.13). The detailed information of the pesticide toxicity loading expressed as aAPTLc showed a difference between the management types. The index for organic (19.91 ± 6.69) was almost twice as high as the integrated vineyards (10.63 ± 14.59) (see also boxplots in Figure S1), but it did not improve model fit according to AIC_c_ ranking. This difference due to the spraying regime was also recognizable in the amount of total pesticide applications, which was about one-third higher in organic (9.69 ± 1.7) than in integrated (6.31 ± 2.24) vineyards. The majority of the applied pesticides were fungicides (synthetic fungicides, sulfur, and copper) (Table 1). Nevertheless, there was a moderate correlation between the aAPTLc and the categorical toxicity rating for *T. pyri* (*R* = 0.62, *p* < 0.001). A similar relationship was also shown between the aAPTLc and the phytoseiid mite densities in relation to the sampling date with a negative moderate correlation (*R* = −0.45, *p* < 0.001). No correlation was found between the categorical toxicity rating for *T. pyri* and the phytoseiid mite densities (*R* = −0.095, *p* = 0.23) (correlations and their significance are shown in Figure S2).

### 3.2. Tydeoid Mites

**Figure 4:**
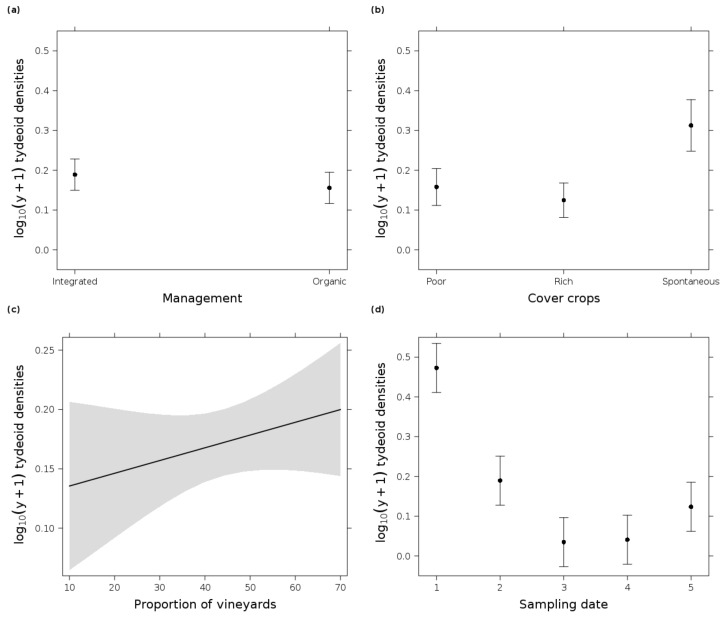
Effect plots of log_10_(y + 1) tydeoid mite densities per 100 cm^2^ vine leaf area in response to (**a**) management (organic and integrated), (**b**) cover crop type (poor = species-poor, rich = species-rich cover crop mixture, and spontaneous = spontaneous vegetation cover), (**c**) proportion of vineyards in the landscape, and (**d**) sampling date in 2019 (1 = 7 May, 2 = 3 June, 3 = 1 July, 4 = 29 July, and 5 = 26 August). (**b**,**d**) originate from the most parsimonious GLM (date + cover crop type); (**a**,**c**) from the third most parsimonious GLM (date + management + cover crop type + proportion of vineyards, Δi = 1.8). Error bars/gray shading indicate 0.95 confidence intervals.

Out of 981 sampled tydeoid mite individuals, *Tydeus goetzi* Schruft was the only identified species. At the beginning of the season, the mean density of *T. goetzi* was 3.5 ± 6.02 SD per 100 cm^2^ leaf area, which decreased to 0.4 ± 0.55 at the end of the season (Figure 2). The most parsimonious GLM (Table 2) indicated significant effects of the sampling date and cover crop type at the field scale. Tydeoid mite densities decreased throughout the season and only increased slightly at the end of the season. Highest densities were found in vineyards with spontaneous vegetation in the inter-row. The next two parsimonious GLMs (Table 2) showed that tydeoid mites were slightly more abundant in integrated compared to organic vineyards and their densities increased with higher proportions of vineyards at the landscape scale (Figure 4).

### 3.3. Phytophagous Mites

**Figure 5:**
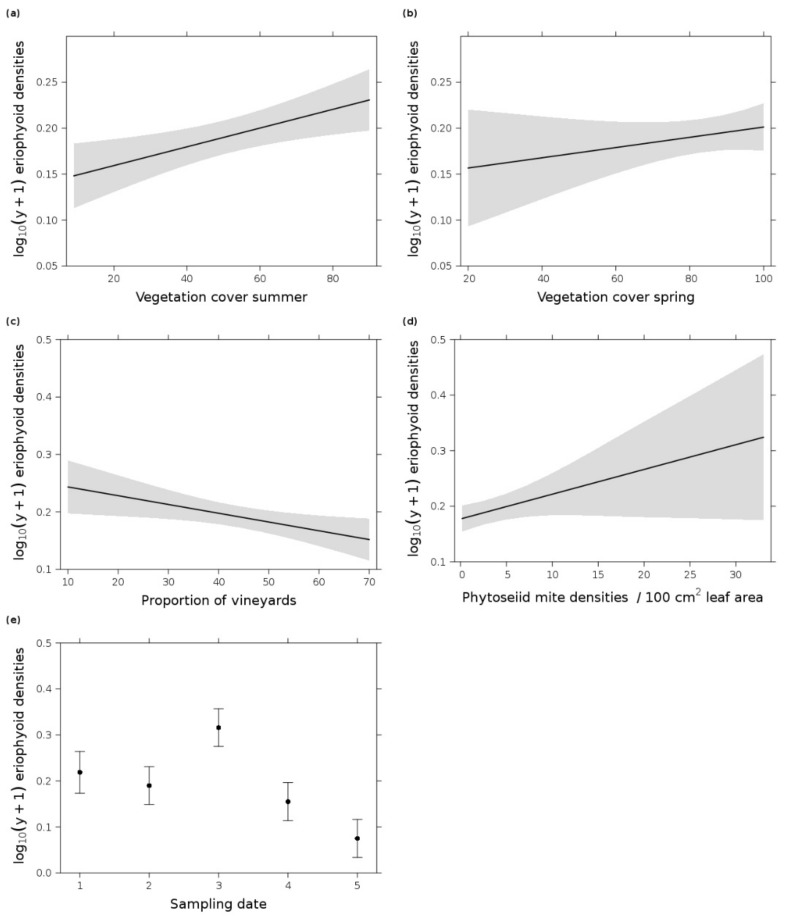
Effect plots of log_10_(y + 1) eriophyoid mite densities per 100 cm^2^ vine leaf area in response to (**a**) vegetation cover in summer, (**b**) vegetation cover in spring, (**c**) proportion of vineyards in the landscape, (**d**) phytoseiid mite densities per 100 cm^2^ leaf area, and (**e**) sampling date in 2019 (1 = 7 May, 2 = 3 June, 3 = 1 July, 4 = 29 July, and 5 = 26 August). (**a**,**c**–**e**) originated from the most parsimonious GLM (date + proportion of vineyards + vegetation cover summer + densities of phytoseiid mites) and (**b**) from the third most parsimonious GLM (date + proportion of vineyards + vegetation cover summer + vegetation cover spring, Δi = 1.97). Error bars/gray shading indicate 0.95 confidence intervals.

Eriophyoid mites, but not spider mites, were present in the grapevine canopy. The species *Colomerus vitis* (Pagenstecher) and *Calepitirimerus vitis* (Nalepa) were present in very low densities at the beginning of the growing season (*Col. vitis*: 0.6 mean ± 0.53 SD per 100 cm^2^ leaf area; *Cal. vitis*: 0.23 ± 0.29) and at the end of the sampling period (*Col. vitis*: 0.03 ± 0.06; *Cal. vitis*: 0.16 ± 0.18) (merged together in Figure 2). Considered together as eriophyoid mites per leaf, only 0–4 mites per leaf (minimum and maximum) occurred throughout the whole sampling season. The population fluctuated over time and they were also absent in some vineyards, especially at the end of the season. The most parsimonious GLM (Table 2) showed that the herbivorous mite density fluctuated over time and increased when the vegetation cover and the phytoseiid mite density increased. Furthermore, at the landscape scale, their densities decreased with higher proportions of vineyards (Figure 5), which was the exact opposite to the phytoseiid mite models. The second most parsimonious GLM (Δi 1.03) (Table 2) contained the same variables but excluding the variable phytoseiid mite density. The third most parsimonious GLM (Δi 1.97, Table 2) contained, in addition to the above-mentioned model, also the vegetation cover in spring, which also increased the herbivorous mite density (Figure 5).

### 3.4. Pollen

**Figure 6:**
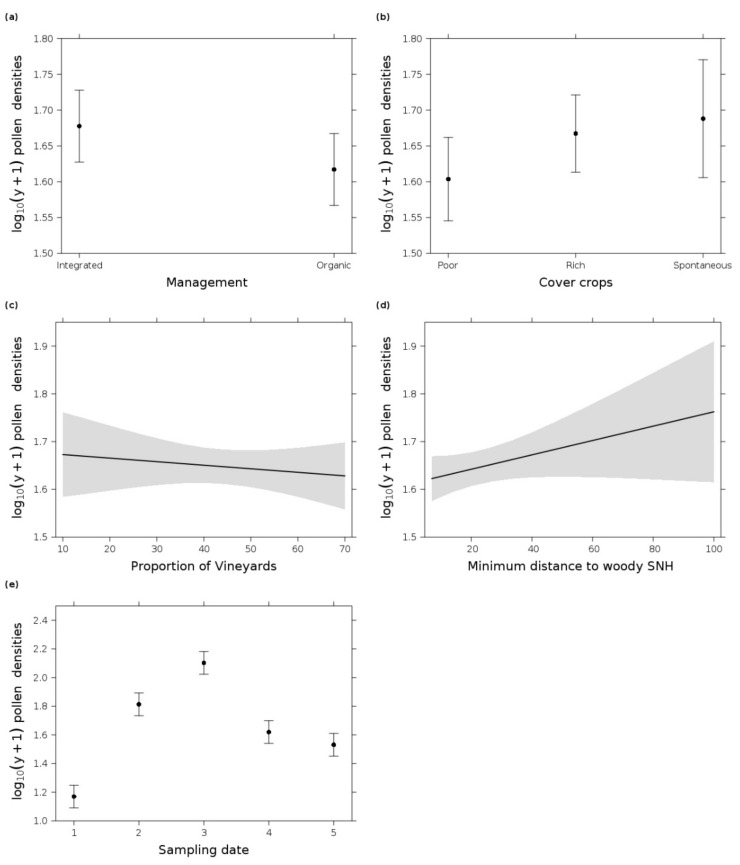
Effect plots of log_10_(y + 1) pollen densities per square centimeter vine leaf area in response to (**a**) management (organic and integrated), (**b**) cover crop type (poor = species-poor, rich = species-rich cover crop mixture, and spontaneous = spontaneous vegetation cover), (**c**) proportion of vineyards in the landscape, (**d**) minimum distance to next woody semi-natural habitats (SNHs), and (**e**) sampling date in 2019 (1 = 7 May, 2 = 3 June, 3 = 1 July, 4 = 29 July, and 5 = 26 August). (**a**,**d**,**e**) originated from most parsimonious GLM (date + management + minimum distance to next woody SNHs), (**b**) from the fifth most parsimonious GLM (date + cover crop type, Δi = 1.49), and (**c**) from the eight most parsimonious GLM (date + management + proportion of vineyards + minimum distance to next woody SNHs, Δi = 1.84). Error bars/gray shading indicate 0.95 confidence intervals.

Pollen grains were always present on vine leaves, and the densities peaked at the beginning of June with up to 138.41 mean ± 58.35 SD pollen grains per square centimeter vine leaf and dropped to 36.06 ± 15.66 pollen grains at the end of the sampling season (Figure 2). The average pollen density across all sampling dates was 63.21 ± 56.83 pollen grains per square centimeter vine leaf. Altogether, 30 different pollen types were identified and the three most frequently found pollen types were found to be derived from the Poaceae family (56.47%), followed by Plantaginaceae (8.3419%) and Pinaceae (7.324%) (Table S3). The GLM analysis showed eight more or less equally parsimonious models, which included field and landscape parameters (Table 2). Overall, pollen density was highest in integrated vineyards and in vineyards with spontaneous vegetation cover. Surprisingly, pollen densities increased further away from woody elements at the landscape scale and slightly decreased when the proportion of vineyards increased (Figure 6). The NMDS showed no distinguishable aggregation of the pollen diversity on vine leaves in relation to cover crops in inter-rows in spring but a lot of vineyards were clearly associated with pollen from Amaranthaceae, Caryophyllaceae, Betulaceae, Juglandaceae, Poaceae, Plantaginaceae, and Pinaceae and with arboreal and non-arboreal pollen. SHDI, the proportion of agricultural land use, and the cover of Amaranthaceae in the vineyard inter-rows were important traits in relation to spring pollen composition (Figure S3). A different trend was recognizable for the summer pollen composition, where only the proportion of SNHs was an important trait. No aggregation of the pollen composition to cover crops was recognizable in summer. It must be considered that the NMDS of the summer pollen diversity had a stress value of 0.24 and should therefore be interpreted with care (Figure S4).

## 4. Discussion

Our findings demonstrated that different vineyard management practices at the field scale and the composition of the surrounding landscape were important factors for mites in the vine canopy. Predatory mite densities benefited from integrated vineyard management with spontaneous vegetation cover. At the landscape scale, predatory mites profited from a higher proportion of surrounding vineyards. The reduced use of pesticides was likely the main factor and may have increased predatory mite densities and also explained the seasonal abundance of the phytoseiid mite species *T. pyri*. In the absence of phytophagous mites, phytoseiid mite populations occurred at low levels throughout the sampling season, which is an important indicator of the effective pest control potential in the investigated vineyards [36]. Neither landscape diversity nor a higher proportion of semi-natural elements affected pollen density, whereas field parameters such as integrated management, species-rich cover crops, or spontaneous vegetation cover may lead to higher pollen densities on vine leaves. Phytoseiid mite densities showed only a very weak response to pollen densities. Regarding phytophagous mites, spider mites were absent in all vineyards, and densities of eriophyoid mites were very low during the whole season. Overall, predatory and phytophagous mite densities were influenced by sampling date in accordance with several other studies [34,35,115,116,117] with a peak for predatory mites at the beginning of May.

### 4.1. Integrated Management and Low Pesticide Use Increased Predatory Mite Densities 

The management type (organic vs. integrated) was one major factor that influenced predatory mite densities. Both phytoseiid and tydeoid mite populations were greater in vineyards under integrated than under organic management. Our findings in Austrian vineyards contrasted with other studies, which showed that phytoseiid mite populations benefited from organic management [48,49]. An explanation for these opposing results could be related to the country-specific pesticide applications, which were higher in organic than integrated vineyards in Austria compared to a recent study in French vineyards with the opposite situation [33]. A possible reason for that difference could be related to the different climatic conditions in each country [18], the local complex of pests and diseases [17], or the greater reliance on prophylactic use of pesticides [31] in conventional viticulture. Similar to Austria, pesticide applications in organic vineyards were higher and phytoseiid densities lower compared to conventional vineyards in Germany and Switzerland [118,119]. The main reason for the higher application frequency in organic vineyards compared to conventional ones in Austria, Germany, and Switzerland is the use of copper and sulfur instead of synthetic fungicides against mildew fungus diseases. Several other studies clearly demonstrated that phytoseiid and tydeoid mite densities were higher in vineyards without pesticide application compared to pesticide-treated vineyards [48,49,120,121]. In Germany, phytoseiid mite populations increased when the fungicide applications were reduced [119,122]. Along the same line, integrated vineyards in this study had less fungicide applications and higher phytoseiid mite densities than organic ones. In this context, the timing of sulfur applications, pre- or post-bloom, affects predatory mite populations, where post-bloom results in the lowest population densities [123]. It is important to look beyond application frequencies and amounts because different active ingredients cause different harmful effects on phytoseiid and tydeoid mites (e.g., mancozeb, meptyldinocap, myclobutanil, paraffinic oil, spinosad, sulfur) [53,54,124,125,126]. The selected aAPTLc index [59] reflects the acute toxicity load on the basis of honeybees as LD_50_ values for all relevant active ingredients due to the problem that not all LR_50_ values were available for phytoseiid mites. Interestingly, the toxicity load was almost twice as high in organic than in integrated vineyards. To cope with the drawback that bees could respond differently to pesticides than predatory mites, the aAPTLc was correlated with the categorical toxicity rating based on *T. pyri*. The results showed that the aAPTLc correlated moderately with the categorical toxicity rating for *T. pyri* and the aAPTLc also correlated moderately negative with the predatory mite densities. In contrast, the categorical toxicity rating for *T. pyri* showed no correlation with the predatory mite densities. We suggest that this could be related to the higher pesticide resistance of *T. pyri* field populations [127] compared to lab strains, which are usually used for ecotoxicological tests [128]. This could be one possible reason why neither aAPTLc nor the categorical toxicity rating based on *T. pyri* were included in the most parsimonious models. Overall, phytoseiid and tydeoid mites benefited from the less intensive use of fungicides in integrated vineyards, which were the most commonly applied pesticides in the studied vineyards. The reduced input of inorganic fungicides such as sulfur and copper seemed to have a positive effect.

### 4.2. The Cover Crop Type Influenced the Mite Populations

The densities of phytoseiid and tydeoid mites and pollen were highest in vineyards with spontaneous vegetation cover. These findings confirmed the crucial role of cover crops or rather, in this case, spontaneous vegetation cover in vineyard inter-rows [27,29]. Spontaneous vegetation consists of plant communities that are adapted to the local conditions with a high proportion of native plants. Two recent studies showed that native cover crops enhanced arthropod diversity [30] and native grasses increased natural enemy abundance [129] in vineyards. Cover crops in general are also important because they provide pollen on vine leaves for phytoseiid [40,43,46,47,130] and tydeoid mites [44,45]. Our results are in line with two other studies, which showed that cover crops were important management measures increasing phytoseiid mite populations [23,131]. This is different for tydeoid mites, Vogelweith and Thiéry [29] discovered that their population were higher in bare soil compared to inter-rows with cover crops because of increased natural enemy populations in cover crops. Another important factor regarding vegetation cover as reservoir for phytoseiid mites is due to the similar seasonal activity pattern of their populations in the inter-row vegetation and on vine leaves [132]. This may enhance the possible dispersal potential of predatory mites from cover crops onto vine leaves, which was already shown for phytoseiid mites on peach [133] and citrus trees [134].

Eriophyoid mite densities were very low (between 0 and 4 mites per leaf) and far beyond reaching any economic threshold level. Standard thresholds for economic injuries for *Cal. vitis* are above 280 mites per leaf [135], and leaves have been shown to develop normally when they were infested with about 20–30 *Col. vitis* [136]. The absence of spider mites could be due to a stable phytoseiid mite population with effective biological control potential [137]. Additionally, the negligible use of insecticides and acaricides in the examined vineyards should also promote high phytoseiid mite abundances that control phytophagous mites [36]. The influence of vegetation cover and phytoseiid mite densities on the phytophagous mite population should be interpreted with care, due to the very low population densities of eriophyoid mites in the current study.

### 4.3. Predatory Mite Densities Benefited from Surrounding Vineyards

Our results showed that higher proportion of vineyards in the landscape were associated with increased predatory mite densities in vineyards. Furthermore, phytoseiid mite densities decreased when SNHs increased. These findings were in contrast with other studies, which showed positive relations between higher landscape diversity or higher shares of SNHs promoting biological control of different pests in vineyards [16,138,139,140]. Several studies demonstrated that SNHs (e.g., hedgerows, maple trees) were an important natural reservoir for phytoseiid mites [34,141], from where they could migrate into nearby vineyards [35,142,143] after their extinction by pesticides or when vineyards were newly established. As Austrian viticultural landscapes are comparably diverse with small-scaled vineyards containing a high proportion of SNHs (10–55%) at the landscape-scale, pesticides (here fungicides) are the most probable limiting factor for predatory mites. The aerial dispersal distance of phytoseiid mites is low and lies between 10 and 200 m through a single or multiple dispersal events during a vegetation season [77,78]. Phytoseiid mites could also disperse between adjacent vineyards, when these vineyards already contained high phytoseiid mite populations [142], which could explain the results of this study. Predatory mite populations seemed to benefit more from adjacent vineyards than from SNHs, probably due to the already established, stable populations and a diverse ecosystem in the studied vineyard inter-rows. The missing effect of SNHs was already shown for phytoseiid mite densities in a recent French study, indicating no effect of landscape complexity [144]. However, conceivable variables that were not measured in our study, the effect of grape varieties [116], the occurrence of mildew fungi [39,41], or the population-specific pesticide resistance of mites [120,127] could also mask the effect of SNHs.

In contrast, eriophyoid mite densities, which were far below economic threshold levels [135,136], decreased with higher proportions of vineyards at the landscape scale.

Therefore, we could not confirm the resource concentration hypothesis [4,5] for eriophyoid mites in vineyards or the natural enemy hypothesis [4,5] for predatory mites. This could be most likely linked to the very low pest mite densities, which masked predator–prey interactions. Phytoseiid mites on vines use, as generalists, a broad spectrum of nutritive food sources (e.g., pollen, fungi) [39] and therefore they can retain in stable populations also in the temporal absence of phytophagous mites. This could be similar for tydeoid mites, which also feed on pollen, fungi [44,45,117], and eriophyoid mites [38].

### 4.4. Low Diversity of Phytoseiids

A diversity survey of phytoseiid species in Austrian vineyards conducted in 1985 in Burgenland close to our study region indicated the occurrence of five species, namely, *Phytoseius bakeri* Chant (relative abundance: 46.52%), *T. pyri* (45.39%), *Amblyseius andersoni* (Chant) (4.20%), *E. finlandicus* (3.81%), and *P. talbii* (0.08%) [145]. These findings showed that the co-dominant species *P. bakeri* was replaced by the dominant *T. pyri* (98.73%), *A. andersoni* also disappeared, and *E. finlandicus* (0.65%) and *P. talbii* (0.62%) were only present in negligible densities in 2019. Similar results were recorded from phytoseiid mite samplings in German vineyards in 2014 [115] and 2018 [119]. Three potential causes may be put forward to explain the current dominance of *T. pyri* in vineyards. (1) This dominance could be related to the pesticide resistance of *T. pyri* populations [120,127], which were released in large numbers as biocontrol agents in the previous 20–30 years in the study region. (2) Due to these frequent releases resulting in higher population densities than other native species, *T. pyri* was able to eliminate other phytoseiid mite species [146]. (3) Climate warming increased the likelihood of heat waves in the last two decades [147], and the dominance of *T. pyri* might be attributed to a high heat-tolerance compared to other phytoseiid species native in Austria. These assumptions, however, should be verified in continuative experiments. *Tydeus goetzi* as the only identified tydeoid mite species in the Austrian study region was also found as the dominant species in Germany in recent studies [115,119].

### 4.5. Factors Influencing Pollen Availability

Pollen is a staple food resource for most phytoseiid [46,47] and tydeoid mites [44,45]. Several field and landscape-scale variables influence pollen densities on vine leaves. We suppose that integrated vineyards exhibited higher pollen densities due to lower pesticide application rates, reducing the possible roll of events from large application droplets or blow off through airblast by the fans of the spraying machinery, which could both remove pollen from the leaves [148]. In comparison to species-rich and species-poor cover crops, spontaneous vegetation supplied most pollen on vine leaves. The landscape was also an important influencing factor and indicated that the distance to woody elements was an essential factor and the vine leaves contained more pollen farther away from these elements. It is likely that pollen was deposited on the vine leaves mostly through aerial dispersal [149]. Hedges and trees therefore might act as pollen filters in comparison to vineyards. For example, a study from Duso et al. [47] showed that the pollen abundance was lower on vine leaves than on leaves from hedgerows, which surrounded the vineyards. Nevertheless, complex landscapes most likely increase pollen densities at the landscape scale [144], which explains why a higher proportion of vineyards leads to a minor decrease of the pollen densities. The NMDS showed no aggregation of the pollen communities in response to cover crop types. As a whole, landscape diversity and viticultural practices were drivers for pollen density and diversity. Due to their high nutritional value for phytoseiid mites, pollen from trees and grasses are especially important [43]. In the current study, grass pollen and different arboreal pollen, e.g., from birch, pine, and walnut, were most abundant. Therefore, pollen supply seems to be sufficient for phytoseiid and tydeoid mites in order to retain in stable populations in the vine canopy throughout the vegetation period.

## 5. Conclusions

The biological control of phytophagous mites is an important part in sustainable viticulture. We concluded that biodiversity at the vineyard and landscape level and viticultural practices such as integrated pest management are crucial for maintaining viable predatory mite populations. The reduction of the amount and frequency of pesticide applications seems to be the most important factor to reduce the harmfulness to non-target organisms. Furthermore, for increasing plant diversity and predatory mite densities, diverse spontaneous vegetation should be favored over low-diversity cover crop mixtures in vineyard inter-rows. Our results show that spontaneous vegetation cover should be more considered in the regulations of the Austrian agri-environmental program, which currently requires participating wine growers in the erosion mitigation measure to seed cover crop mixtures. The aAPTLc index is useful to quantify and evaluate the toxicity load for non-target organisms as it extends beyond simple treatment frequency indices. This study shows the great potential of low pesticide use and spontaneous vegetation cover for maintaining pest control services in viticultural landscapes.

## Figures and Tables

**Table 1:** Summary of management-related numeric explanatory variables (mean ± SD): pesticide use and inter-row vegetation cover split between organic (*n* = 16) and integrated (*n* = 16) vineyards. The number of pesticide applications (total) is subdivided into a more precise list of applications of different pesticides. For further information of the used active ingredients, see Table S1.

Management-Related Numeric Explanatory Variables	Organic	Integrated
aAPTLc (area-related acute pesticide contact toxicity loading)	19.91 ± 6.69	10.63 ± 14.59
Categorical toxicity rating for *Typhlodromus* *pyri*	14.65 ± 3.57	13.19 ± 5.13
Number of pesticide applications (total)	9.69 ± 1.7	6.31 ± 2.24
Insecticide applications	0 ± 0	0.25 ± 0.45
Acaricide applications	0.25 ± 0.45	0.38 ± 0.5
Synthetic fungicide applications	0 ± 0	5.79 ± 2.58
Sulfur applications	9.19 ± 1.6	4.13 ± 1.84
Copper applications	8.88 ± 1.84	1.25 ± 0.83
Potassium bicarbonate applications	4.44 ± 2.76	0 ± 0
Vegetation cover in spring (%)	82.38 ± 18.94	80.84 ± 19.51
Vegetation cover in summer (%)	61.70 ± 22.26	40.36 ± 24.78

**Table 2:** AIC_c_ (second-order Akaike Information Criterion) values for model selection of the response variables, out of 42 different generalized linear models (GLMs), Δi = difference between AIC_c_ to the next best model. SNHs = semi-natural habitats.

Response Variable	Best Model	AIC_c_	Δi	Adjusted *R*^2^
Phytoseiid mite densities	null model	37.22	-	-
	**date + management + cover crop type + proportion of vineyards + proportion of total SNHs**	−45.87	0.0	0.44
	date + management + cover crop type + proportion of vineyards + proportion of total SNHs + pollen total	−43.74	2.13	0.44
Tydeoid mite densities	null model	9.22	-	-
	**date + cover crop type**	−90.55	0.0	0.49
	**date + management + cover crop type**	−89.71	0.84	0.49
	**date + management + cover crop type + proportion of vineyards**	−88.75	1.80	0.49
	date + management + cover crop type + proportion of total SNHs	−87.97	2.58	0.49
Eriophyoid mite densities	null model	−158.21	-	-
	**date + proportion of vineyards + vegetation cover summer + densities of phytoseiid mites**	−221.32	0.0	0.36
	**date + proportion of vineyards + vegetation cover summer**	−220.29	1.03	0.35
	**date + proportion of vineyards + vegetation cover summer + vegetation cover spring**	−219.35	1.97	0.35
	date + vegetation cover summer	−218.20	3.12	0.34
Pollen densities	null model	150.85	-	-
	**date + management + minimum distance to the next woody SNHs**	−10.43	0.0	0.65
	**date + management**	−10.04	0.39	0.65
	**date + minimum distance to the next woody SNHs**	−9.72	0.71	0.65
	**date**	−9.53	0.90	0.64
	**date + cover crop type**	−8.94	1.49	0.65
	**date + management + cover crop type + minimum distance to the next woody SNHs**	−8.64	1.79	0.65
	**date + management + cover crop type**	−8.60	1.83	0.65
	**date + management + proportion of vineyards + minimum distance to the next woody SNHs**	−8.59	1.84	0.65
	date + management + proportion of total SNHs + minimum distance to the next woody SNHs	−8.34	2.09	0.65

## Data Availability

The data presented in this study are openly available in Zenodo at: http://doi.org/10.5281/zenodo.4450163.

## Supplementary Materials

The following are available online at https://www.mdpi.com/2075-4450/12/2/180/s1: Table S1. Summary of the active ingredients of different types of pesticides applied in the investigated vineyards in 2019. Table S2: Mean and standard deviation (SD) of the landscape parameters in 2019. Table S3: List of all pollen types found on vine leaves during the sampling period of 2019. Figure S1: Boxplots of the different pesticide indexes of organic and integrated vineyards. Figure S2: Spearman’s correlation plots. Figure S3: NMDS (non-metric multidimensional scaling) plot displaying the ordination of the pollen types in spring 2019. Figure S4: NMDS (non-metric multidimensional scaling) plot displaying the ordination of the pollen types in summer 2019.
