# Supplementary material for: Unexpected Effects of Local Management and Landscape Composition on Predatory Mites and Their Food Resources in Vineyards
Source: Insects. 2021 Feb 19;12(2):180. doi: 10.3390/insects12020180 (PMC7922120; doi:10.3390/insects12020180)
Supplement: Supplementary file 1 [file insects-12-00180-s001.pdf]

insects

MDPI

Unexpected Effects of Local Management and Landscape Composition on Predatory Mites and Their Food Resources in Vineyards

Stefan Möth [1,*], Andreas Walzer [1], Markus Redl [1], Božana Petrović [1], Christoph Hoffmann [2] and Silvia Winter [1]

Table S1. Summary of the active ingredients of different types of pesticides applied in the investigated vineyards in 2019 split between organic (n=16) and integrated vineyards (n = 16).

Type of pesticide	Active ingredient	
	Organic management	Integrated management
Fungicide	Copper hydroxide	Copper hydroxide
Fungicide	Potassium bicarbonate	
Fungicide	Copper sulphate	Copper sulphate
Fungicide	Sulphur	Sulphur
Fungicide		Benalaxyl-M
Fungicide		Benthiavalicarb
Fungicide		Boscalid
Fungicide		Copper oxychloride
Fungicide		Cyazofamid
Fungicide		Cyflufenamid
Fungicide		Cymoxanil
Fungicide		Difenoconazol
Fungicide		Dimethomorph
Fungicide		Dithianon
Fungicide		Fluopyram
Fungicide		Fluopicolide
Fungicide		Fluxapyroxad
Fungicide		Folpet
Fungicide		Fosetyl-Aluminium
Fungicide		Iprovalicarb
Fungicide		Kresoxim-methyl
Fungicide		Mancozeb
Fungicide		Meptyldinocap
Fungicide		Metrafenon
Fungicide		Myclobutanil
Fungicide		Oxathiapiprolin
Fungicide		Proquinazid
Fungicide		Potassium phosphonates
Fungicide		Pyriofenon
Fungicide		Quinoxyfen
Fungicide		Spiroxamin
Fungicide		Tebuconazole
Fungicide		Tetraconazol
Fungicide		Trifloxystrobin
Fungicide		Zoxamid
Acaricide	Paraffin oil	Paraffin oil
Insecticide		Spinosad
Insecticide		Indoxacarb

Table S2. Mean and standard deviation (SD) of the landscape parameters 2019 used for statistical analysis across all landscape circles (n = 64). SNHs = semi-natural habitats, SHDI = Shannon's landscape diversity index.

**Landscape parameters**	**mean ± SD**	**min**	**max**
Woody SNHs (%)	13.45 ± 15.25	1.28	51.05
Total SNHs (%)	29.37 ± 13.87	10.4	55.47
Total agricultural area (%)	60.51 ± 16.83	28.58	82.96
Vineyards (%)	44.32 ± 16.94	14.94	69.33
SHDI	1.49 ± 0.23	1.11	2.03
Minimum distance to the next woody SNHs (m)	23.45 ± 18.72	9.43	89.87

Table S3. List of all pollen types found on vine leaves during the sampling period 2019 in percentage (%) for each taxa in relationship to the total pollen grains/cm² vine leave, in spring (7. May and 3. June) and summer (1., 29. July and 26. August). Pollen which was not identifiable at a specific taxon: Arboreal pollen which was not identifiable at family level (AP), non-arboreal pollen which was not identifiable at family level (NAP), not able to identify (NA).

**Pollen types**	**Total (%)**	**Spring (%)**	**Summer (%)**
Poaceae	56.47	13.3419	43.1281
Plantaginaceae	8.3419	1.6781	6.6638
Pinaceae	7.324	6.1804	1.1436
Asteraceae	7.2449	0.2819	6.963
Moraceae and Urticaceae	6.1486	1.1212	5.0274
Amaranthaceae and Caryophyllaceae	4.6323	0.2433	4.389
Vitis	2.0527	0	2.0527
Arboreal pollen (AP)	1.8349	1.8324	0.0025
Not able to identify (NA)	1.4603	0.705	0.7553
Betulaceae	0.9694	0.9563	0.0131
Non-arboreal pollen (NAP)	0.8767	0.8344	0.0423
Tilia	0.6141	0.0025	0.6116
Caryophyllales	0.4219	0.038	0.3839
Brassicaceae	0.4212	0.3671	0.0541
Ranunculaceae	0.2924	0.2806	0.0118
Juglandaceae	0.2688	0.2445	0.0243
Campanulaceae	0.1512	0.1288	0.0224
Apiaceae	0.1188	0.0044	0.1144
Impatiens	0.0778	0	0.0778
Rubiaceae	0.0697	0	0.0697
Plumbaginaceae	0.0678	0.0635	0.0043
Fagaceae	0.0367	0.0367	0
Salicaceae	0.0361	0.033	0.0031
Rumex	0.0131	0.0131	0
Lamiaceae	0.0187	0	0.0187
Fabaceae	0.0137	0	0.0137
Polygonaceae	0.0044	0	0.0044
Rosaceae	0.0044	0.0044	0
Aesculus	0.0031	0.0031	0
Cupressaceae	0.0031	0.0031	0
Sorbus	0.0031	0	0.0031
Typhaceae	0.0031	0	0.0031
Ericaceae	0.0012	0	0.0012

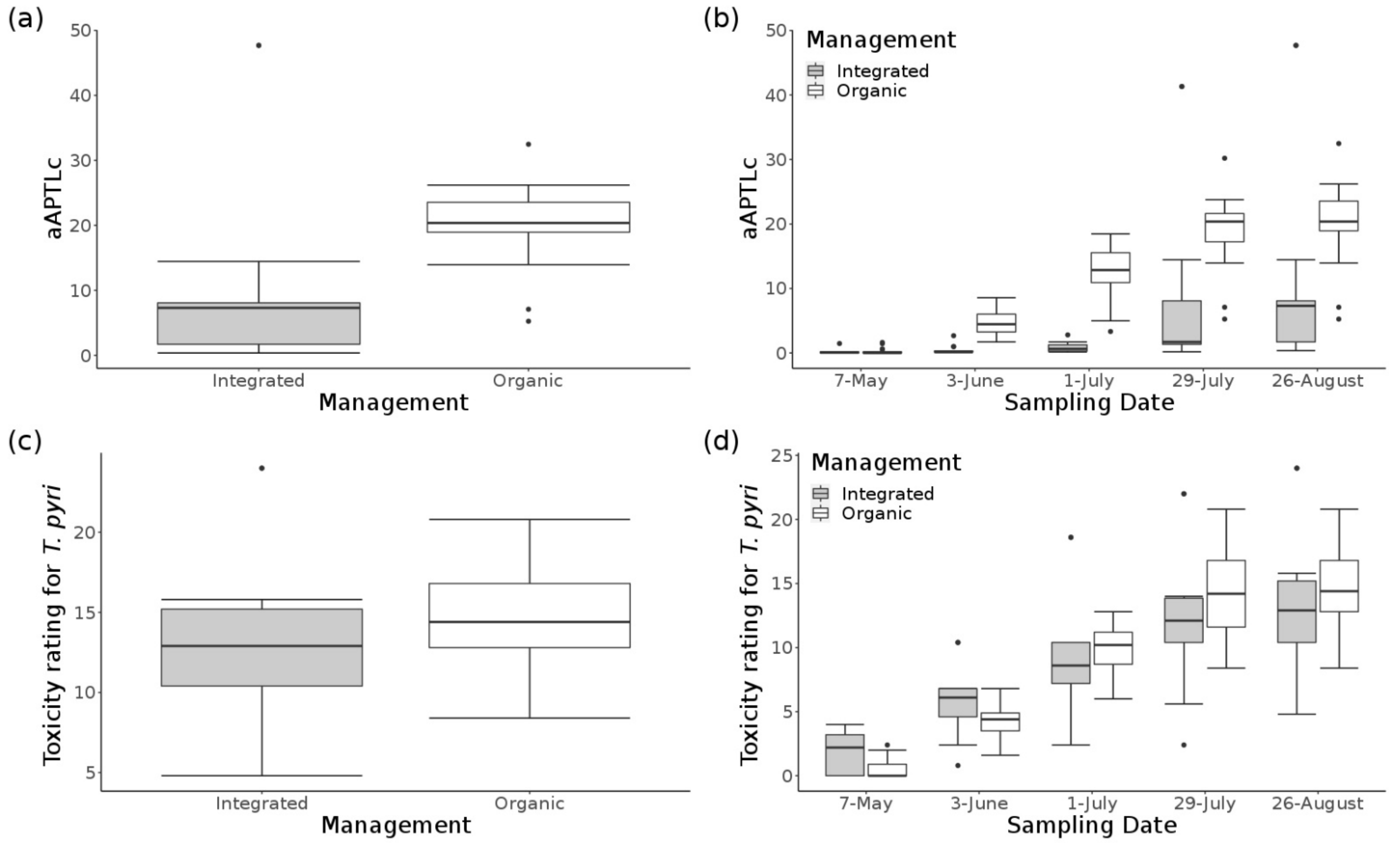

Figure S1. Boxplots of the different pesticide indexes of organic and integrated vineyards from 2019: (**a**) aAPTLc index (area-related acute pesticide contact toxicity loading) in total, (**b**) aAPTLc index aggregated according to the sampling dates, (**c**) the categorical toxicity rating for *Typhlodromus pyri* Scheuten in total and (**d**) the categorical toxicity rating for *T. pyri* aggregated according to the sampling dates. Dots represent outliers.

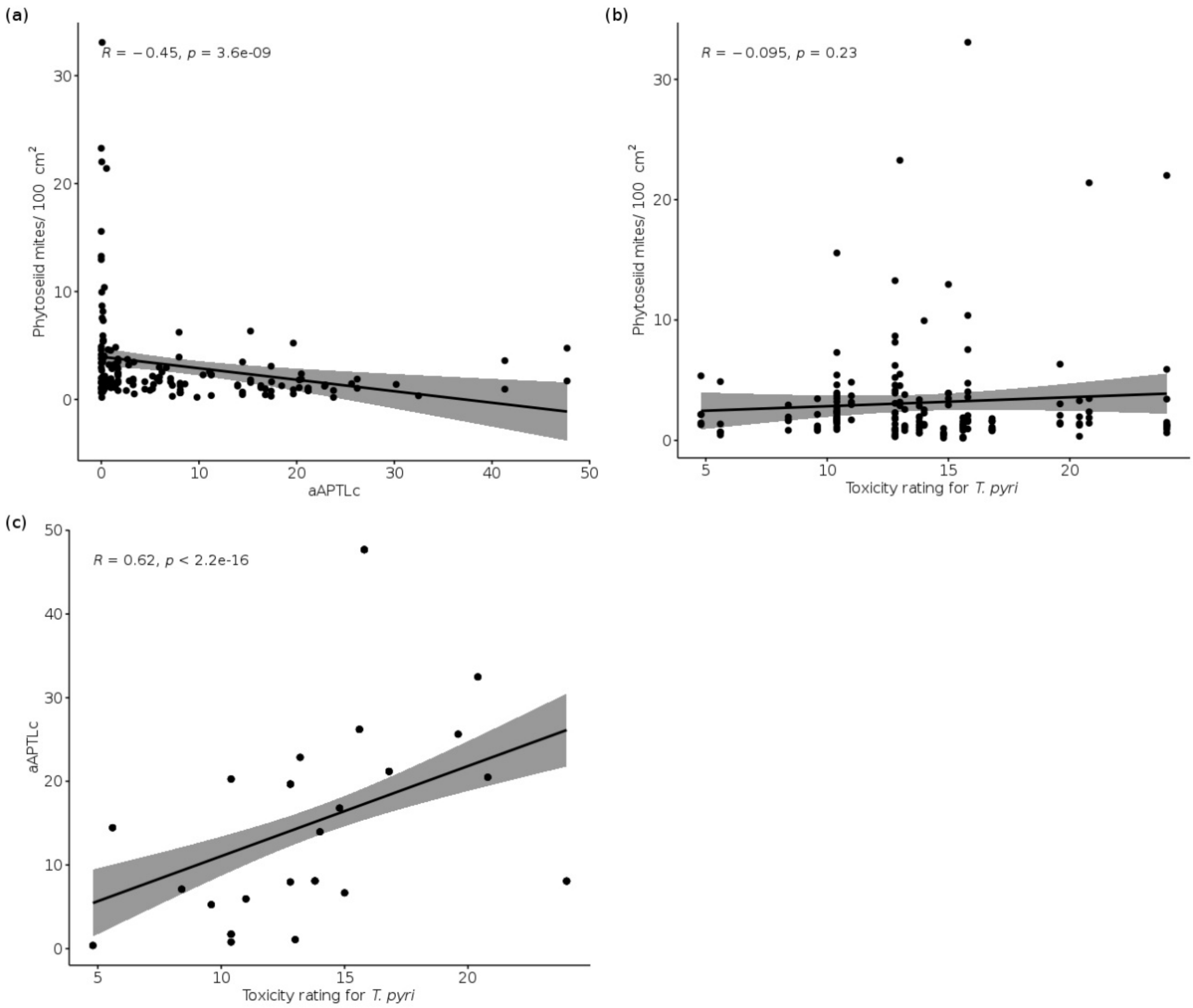

Figure S2. Spearman's correlation plots: (a) aAPTLc (area-related acute pesticide contact toxicity loading) correlated with phytoseiid mite densities per 100 cm² vine leaf area in relation to the sampling date, (b) categorical toxicity rating for *T. pyri* correlated with phytoseiid mite densities per 100 cm² vine leaf area and (c) categorical toxicity rating for *T. pyri* correlated with aAPTLc. R-values and *p*-values for each correlation are shown in the right corner of each corresponding plot.

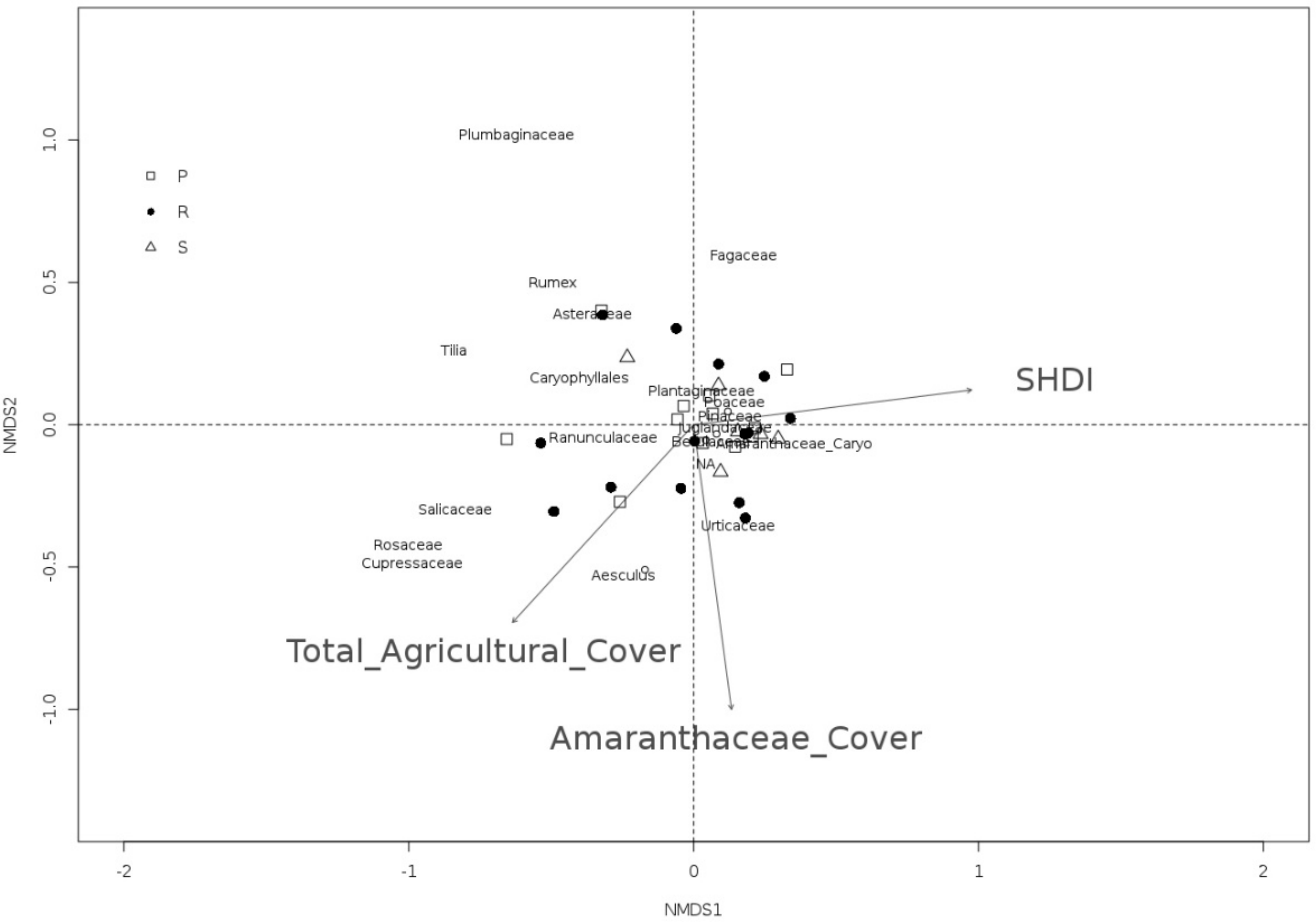

Figure S3. NMDS (Non-metric multidimensional scaling) plot displaying the ordination of the pollen types in spring 2019 (sampling date: 7. May and 3. June) with their associated cover crops (squares: P = species-poor; black dots: R = species-rich cover crop mixtures; triangles: S = spontaneous vegetation). The vectors show significant traits at the landscape and field scale: Shannon's landscape diversity index = SHDI; proportion of total agricultural cover = Total_agricultural_cover; inter-row vegetation cover of Amaranthacea = Amaranthaceae_Cover; in relation to the community composition of the pollen on vine leaves. NA = not able to identify, AP = arboreal pollen, NAP = non-arboreal pollen, Amaranthaceae_Caryo = Amaranthaceae and Caryophyllaceae, Urticaceae = Urticaceae and Moraceae. Stress value = 0.17.

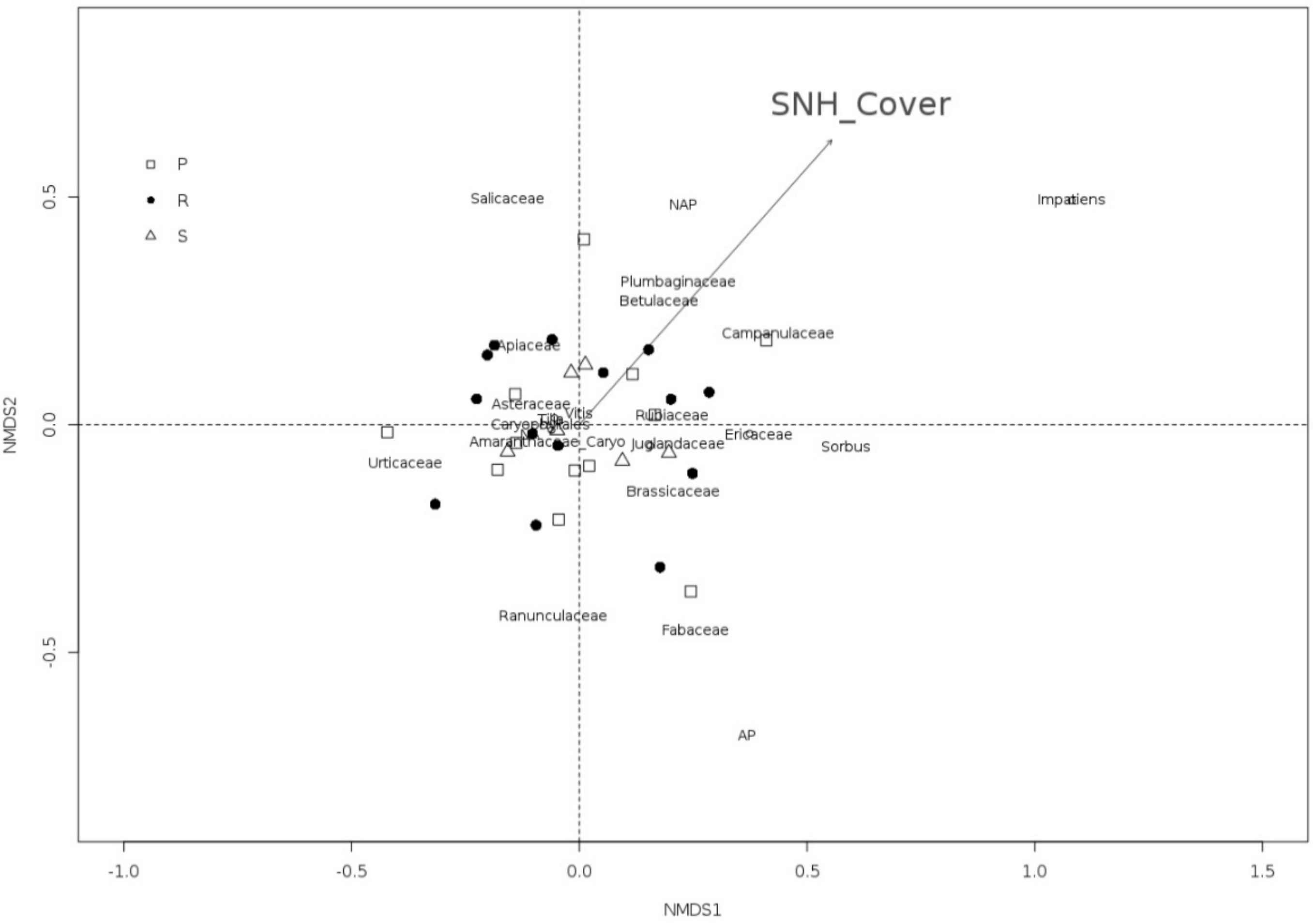

Figure S4. NMDS (Non-metric multidimensional scaling) plot displaying the ordination of the pollen types in summer 2019 (sampling date: 1., 29. July and 26. August) with their associated cover crops (squares: P = species-poor; black dots: R = species-rich; triangles: S = spontaneous vegetation). The vector shows significant traits at the landscape scale: proportion of semi-natural habitats (SNHs) = SNH_Cover; in relation to the community composition of the pollen on vine leaves.. NA = not able to identify, AP = arboreal pollen, NAP = non-arboreal pollen, Amaranthaceae_Caryo = Amaranthaceae and Caryophyllaceae, Urticaceae = Urticaceae and Moraceae. Stress value = 0.24.
